# Synergetic Enhancement of Tumor Double-Targeted MRI Nano-Probe

**DOI:** 10.3390/ijms23063119

**Published:** 2022-03-14

**Authors:** Nikita Yabbarov, Elena Nikolskaya, Maria Sokol, Mariia Mollaeva, Margarita Chirkina, Irina Seregina, Mikhail Gulyaev, Yury Pirogov, Rem Petrov

**Affiliations:** 1 N. M. Emanuel Institute of Biochemical Physics of Russian Academy of Sciences, 119334 Moscow, Russia; elenanikolskaja@gmail.com (E.N.); mariyabsokol@gmail.com (M.S.); mollaevamariia@gmail.com (M.M.); fom.marg@mail.ru (M.C.); 2 Department of Chemistry, Lomonosov Moscow State University, 119991 Moscow, Russia; sereginairinaf@mail.ru; 3 Faculty of Fundamental Medicine, Lomonosov Moscow State University, 117192 Moscow, Russia; gulyaev@physics.msu.ru; 4 Faculty of Physics, Lomonosov Moscow State University, 119991 Moscow, Russia; yupi937@gmail.com; 5 Shemyakin-Ovchinnikov Institute of Bioorganic Chemistry, 117997 Moscow, Russia; rem.petrov.30@mail.ru

**Keywords:** alpha-fetoprotein, dendrimer, targeted delivery, selectin, vascular extravasation, double-targeting

## Abstract

The conventional targeted delivery of chemotherapeutic and diagnostic agents utilizing nanocarriers is a promising approach for cancer theranostics. Unfortunately, this approach often faces hindered tumor access that decreases the therapeutic index and limits the further clinical translation of a developing drug. Here, we demonstrated a strategy of simultaneously double-targeting the drug to two distinct cites of tumor tissue: the tumor endothelium and cell surface receptors. We used fourth-generation polyamideamine dendrimers modified with a chelated Gd and functionalized with selectin ligand and alpha-fetoprotein receptor-binding peptide. According to the proposed strategy, IELLQAR peptide promotes the conjugate recruitment to the tumor inflammatory microenvironment and enhances extravasation through the interaction of nanodevice with P- and E-selectins expressed by endothelial cells. The second target moiety—alpha-fetoprotein receptor-binding peptide—enhances drug internalization into cancer cells and the intratumoral retention of the conjugate. The final conjugate contained 18 chelated Gd ions per dendrimer, characterized with a 32 nm size and a negative surface charge of around 18 mV. In vitro contrasting properties were comparable with commercially available Gd-chelate: r1 relaxivity was 3.39 for Magnevist and 3.11 for conjugate; r2 relaxivity was 5.12 for Magnevist and 4.81 for conjugate. By utilizing this dual targeting strategy, we demonstrated the increment of intratumoral accumulation, and a remarkable enhancement of antitumor effect, resulting in high-level synergy compared to monotargeted conjugates. In summary, the proposed strategy utilizing tumor tissue double-targeting may contribute to an enhancement in drug and diagnostic accumulation in aggressive tumors.

## 1. Introduction 

Cancer represents a complex disease type that causes substantial mortality in modern society. Despite the plentitude of traditional drugs (e.g. daunorubicin, paclitaxel, etc.) and the development and clinical application of novel monoclonal antibodies (mABs) and small-molecule inhibitors, modern therapy still demands safe and effective anticancer therapeutics and diagnostics. 

Nanoparticle (NP)-based drug delivery systems continue to attract enormous attention as important vehicles in cancer therapy [1]. Due to their small size, variability of preparations and modifications, and specific tumor physiology, NPs are widely applied to enhance anticancer drug accumulation in tumor tissues and reduce side effects [2,3]. Block copolymers [4], dendritic polymers [5], silica-based particles and fibers [6,7], and metal-organic frameworks [8] represent a significant portion of the matrixes applied in nanovehicles’ design. NPs possess a convenient structure for mono and multidrug loading, which allows for the construction of simple and complicated systems: multidrug-loaded NPs, theranostics, etc. [9,10,11,12,13]. Rigorous drug selection and optimal formulation methods may augment antitumor action greatly and reduce side effects [14,15]. 

Unfortunately, passive targeting via enhanced permeability and retention effect (EPR) is often insufficient, so researchers additionally apply various targeting ligands to ensure a sufficient accumulation and antitumor effect, such as antibodies, growth factors, transferrin, alpha-fetoprotein, cytokines, folic acid, low-density lipoprotein, tumor proteases, and tumor matrix proteins [16,17,18,19]. 

Today, along with monotargeted vehicles, multi-targeting strategies have become more popular. Several recent reports describe the formulation of dual targeting systems that utilize two or more vector moieties to deliver drugs into tumors: markers of distinct tissues (e.g., folate, which facilitates internalization into cancer cells, and transferrin, which provides blood–brain barrier penetration) [20] and markers of different cells (tri- or bi-specific antibodies that promote the interaction of lymphocytes with tumor cells) [21]. The multiple targeting may represent the different moieties applied at different times, e.g., the pretreatment of tumor-bearing mice with junction opener proteins (JO-4) before targeted vehicle injection or gene silencing with synchronic receptor blockade [22,23]. Small molecules (e.g., nitroglycerin as an EPR effect enhancer) or the application of physical methods (e.g., focused ultrasound for temporary blood–brain barrier penetration enhancement) are also used for targeted delivery augmentation [24,25]. The general advantage of these multiple targeting systems over traditional systems is the significant treatment efficiency and enhanced target specificity [26]. Unfortunately, complex tumor physiology, fast clonal expansion, drug resistance development, and metastasis expansion rarely allow scientists to select the appropriate combination of targets, especially ones that display a strong synergy [27]. In this study, we focused on the design of a relatively simple system carrying two tumor-targeting moieties and evaluated the influence of the presence of the second vector on the tumor-specific accumulation in vitro and in vivo.

One of the most widespread and attractive tumor-targeting ligands is alpha-fetoprotein (AFP) and its analogs, which are able to be internalized specifically by a range of tumor cells and tissues. Various types of tumors (osteosarcoma, neuro- and glioblastoma, melanoma, lung carcinoma, breast adenocarcinoma, etc.) possess a significantly enhanced AFP receptor expression [28]. Normal tissues demonstrate a lack of or low expression level of this receptor. AFP and its receptor-binding fragments may provide a favorable drug distribution and high accumulation in tumor tissue, which makes their application attractive as vector molecules [29,30,31]. Multiple studies have demonstrated an enhanced antitumor effect of AFP or AFP-receptor binding fragments-decorated nanosystems in vitro and in vivo [32,33,34]. 

Another widespread characteristic feature of tumor tissue is an inflammatory microenvironment. Resting endothelial cells express selectins at extremely low levels. However, inflammatory cytokines such as TNFα and IL-1β strongly enhance their expression via transcriptional regulation [35]. Normally, selectins facilitate leukocytes low-affinity rolling along the endothelium to the specific location for further strong integrin-mediated interactions at the inflammation sites. The same mechanism emerges during tumor development, when cancer cells produce cytokines to attract innate immune cells and form an immunosuppressive microenvironment [36]. Inflamed endothelial cells in tumor tissues are an attractive target for delivery. Previous reports describe several types of similar selectin-targeted delivery systems: lipid and polymer-based NPs conjugated with anti-E-selectin ABs [37,38,39]. Low molecular ligands of selectins represented with syalil Lewis X tetrasaccharide and its analog are also widely utilized as targeting moieties in delivery systems [40,41]. However, sLeX availability is hampered by the complexity and difficulty of its synthesis. IELLQAR peptide is a synthetic selectin ligand that is utilized for tumor vasculature targeting [42,43]. 

Thus, regarding the aforementioned data, we chose two peptides as tumor-targeting moieties, KQEFLIN—AFP receptor-binding peptide and IELLQAR—P-selectin-biding peptide, which were immobilized on the surface of polyamideamine dendrimers. 

We demonstrated an approach employing distinct binding moieties of tumor tissue for drug delivery. The different location and expression levels of the selectins (tumor endothelial cells) and AFP receptors (tumor cells) at the same tumor nodes allowed us to improve targeted delivery on the nanovehicle compared to monotargeted conjugates. The present and aforementioned studies describing multitargeting strategies resemble the widespread treatment strategies of bacterial infections (combinations of antibiotics), cancer (combinations of anticancer drugs), and viral infections (e.g., highly effective combinations of targeted anti HIV/AIDS drugs). Thus, we expect that the described study will provide not only information about the accumulation efficacy of this system in tumors, but also help researchers choose the appropriate carrier, drug, or vector and evaluate the worthiness of the delivery approach that will be applied. 

## 2. Materials and Methods

### 2.1. Materials

Polyamidoamine dendrimer (PAMAM 4th generation) was purchased from Dentritech (Midland, MI, USA). NHS-PEG-Mal (MW 5 kDa), and NHS-PEG-OCH_3_ (MW 5 kDa) purchased from RuixiBiotech (Xian, China). Solid-phase methods were applied to synthesize IELQARCRR and KQEFLINCRR peptides. D_2_O, arsenazo III, GdCl_3_, reduced glutathione (GSH), 4,4′-Dithiodipyridine (DTDP), and 3-(4,5-Dimethylthiazol-2yl)-2,5,diphenyltetrazolium bromide (MTT reagent) were purchased from Sigma-Aldrich (St. Louis, MO, USA). p-SCN-Bn-DTPA was purchased from Macrocyclics (Plano, TX, USA). DMEM and FBS were purchased from Gibco (Waltham, MA, USA). Hoechst 33342, Fluorescein isothiocyanate (FITC), and Cy5.5 N-hydroxysuccinimide (Cy5.5-NHS) were purchased from Lumiprobe (Moscow, Russia). Penicillin–streptomycin and gentamycin were purchased from PanEco (Moscow, Russia). NaHCO_3_, NH_4_CH_3_CO_2_, and PBS were purchased from Chimmed (Moscow, Russia). 10 kDa MWCO dialysis bags were purchased from CelluSep (Seguin, TX, USA). 

### 2.2. PAMAM G4 Derivatives Synthesis

PAMAM G4 dendrimers were labeled with p-SCN-Bn-DTPA and FITC (in vitro imaging) or Cy5.5 (in vivo imaging). A total of 5 µM of PAMAM G4 was dissolved in 10 mL of 125 mM NaHCO_3_, mixed with 10 molar equivalents of p-SCN-Bn-DTPA, stirred at room temperature (RT) for 24 h, purified by dialysis (2 kDa MWCO membrane) against aqueous solution of 125 mM NaHCO_3_ and then against deionized water, and finally freeze-dried. The DTPA content was analyzed quantitatively using the previously described spectrophotometric method [44]. G4-DTPA metallation was accomplished in 100 mM of ammonium acetate buffer pH 5.5; 1.5 molar equivalents of GdCl_3_ was added to DTPA and the reaction mixture was incubated at 37 °C for 2 h. The amount of bound Gd was measured applying ICP-MS analysis and using arsenazo III [45].

The labeling of G4-DTPA with FITC or Cy5.5 was performed in 100 mM of NaHCO_3_ over 15 h at RT; the amount of dye bound after purification by dialysis was measured at λ_495_ for FITC and λ_680_ for Cy5.5. 

NHS-PEG-Mal (50 µM) and NHS-PEG-OCH_3_ (110 µM) were mixed with PAMAM G4 (5 µmol) in 5 mL of PBS. The mixture was stirred at 25 °C for 24 h and twice dialyzed against 1 L of deionized water during 48h. The final solution was freeze-dried.

### 2.3. PAMAM G-4 PEG-Mal/OCH_3_ Coupling with IELLQARCRR and KQEFLINGCRR 

IELLQARCRR (5 µM) and KQEFLINGCRR (5 µM) were mixed separately with G4-PEG-Mal/OCH_3_ (1 µM) in 15 mL PBS, stirred under an argon atmosphere at RT for 24 h, dialyzed twice against 1 L deionized water for 48 h, and then freeze-dried. Below, we used G4-DTPA-PEG-Pep abbreviation for the double-targeted conjugate containing two peptides, and G4-DTPA-PEG-KQ or G4-DTPA-PEG-IEL for the designation of corresponding mono-targeted conjugate labeled with one peptide. 

### 2.4. Characterization

^1^H NMR spectra of all intermediate and final polymer products were recorded at 25 °C on a Bruker Avance III HD 500 Mhz using D_2_O as the solvent and a sample concentration of 5 mg/mL [46].

The Gd content was analyzed with ICP-MS (Agilent 7500C, Agilent Technologies, Tokyo, Japan) [47]. The sample introduction system consisted of a robust Babington nebulizer with a Scott spray chamber (Agilent Technologies, Tokyo, Japan) cooled by a Peltier element (2 °C). The data were acquired and processed with the ICP-MS ChemStation (Agilent Technologies, version G1834B; Santa Clara, CA, USA) software package. For sample dilution, the mixture of 0.1% Triton X-100—1% HNO_3_ was used. The same solution was used as the mobile phase in the mass spectrometer.

The terminal maleimide as well as IELLQARCRR and KQEFLINGCRR residues were quantified after G4-DTPA-PEG conjugation with GSH excess followed by unreacted glutathione colorimetric analysis with DTDP at λ = 324 nm (Ɛ_324_ of DTDP = 19,800 M^−1^cm^−1^) [48]. Briefly, 0.1 mL G4-DTPA-PEG, G4-DTPA-PEG-KQ or G4-DTPA-PEG-IEL in a concentration range of 1–20 µM (according to G4 concentration) was mixed with 0.5 mL of 50 µM GSH in 10 mM PBS at pH 7.4, incubated at 37 °C for 40 min, mixed with 0.25 mL of 120 µM DTDP in 10 mM PBS at pH 7.4, and incubated at room temperature for 2 min; unreacted GSH contributed to absorbance at 324 nm after reaction with DTDP and was used for maleimide termini quantification.

The size distribution of the derivatives was determined by dynamic light scattering (DLS) in deionized water, while the zeta-potential was determined by electrophoretic light scattering in 10 mM of NaCl (Nano-ZS ZEN 3600, Malvern-Instruments, Worcestershire, UK) at a sample concentration of 1 mg/mL at 25 °C [49].

The morphology of the dried polymers was analyzed using a transmission electron microscope (TEM) (Talos F200i S/TEM (Thermo Scientific, Waltham, Massachusetts, US). Samples were diluted with water to a concentration of 1 mg/mL, dispersed onto 3 mm carbon-coated Cu grids, and air-dried.

### 2.5. In Vitro Internalization Assay

The intracellular uptake was analyzed by flow cytometry and confocal laser scanning microscopy (CLSM). SKOV-3 cells were maintained in DMEM supplemented with 5% FBS and 50 µg/mL gentamycin. Cells were incubated with corresponding conjugates labeled with FITC for 1 h and washed with PBS. The intracellular FITC fluorescence was analyzed using a Dako CyAn Flow Cytometer equipped with a 488 nm laser (Beckman Coulter, Brea, CA, USA). For CLSM analysis, cells were seeded onto 24-well plates equipped with round glass slides and maintained in corresponding media. Cells were incubated with conjugates labeled with FITC for 2 h, washed with PBS, and fixed in 2% paraformaldehyde. Nuclei were stained with Hoechst33342. Cells were analyzed with a Zeiss Cell Observer Z1 confocal laser scanning microscope and images were acquired with the AxioVision (Germany) and Imaris (Bitplane AG, Zürich, Switzerland) software.

### 2.6. Cytotoxicity Assay

In vitro cytotoxicity of the conjugates was evaluated applying the MTT assay. Human ovarian adenocarcinoma SKOV-3 cells were plated on 96-well plates and incubated for 24 h in a corresponding medium at a density of 5000 cells per well at 37 °C under humidified atmosphere of 5% CO_2_. The next day, the medium was supplemented with G4 conjugates or G4 in a concentration range of 500—3.9 µM dissolved in DMEM. After 72 h of incubation, cell survival was determined using standard MTT assay: 50 μL MTT in DMEM (1 mg/mL) was added into each well. After cell incubation for 4 h at 37 °C, the medium was removed and precipitated formazan crystals were dissolved in 100 μL DMSO. Next, the absorption intensity of formazan was measured at 540 nm on a microplate reader. Cell viability was determined as a percentage of the untreated control. The survival values were normalized to an untreated control [50].

### 2.7. MR Imaging

MRI studies were performed on a 7.05T MRI scanner Bruker BioSpec 70/30 USR with a ParaVision v.5.1 software. A birdcage of 72 mm internal diameter was used as a transceiver.

The phantoms were the test tubes (eppendorfs of 2 mL volume) filled with substances (Magnevist^®^ or G4-DTPA-PEG-Pep) diluted in distilled water in different Gd concentration: 25 mM, 12.5 mM, 6.25 mM, 3.125 mM, and 1.625 mM. One more phantom filled with distilled water was used as a reference. Thus, there were 11 phantoms in total.

To compare the contrast properties of the substances (G4-DTPA-PEG-Pep and Magnevist^®^), we determined their relaxivities *r*_1_ and *r*_2_, which reflect a linear correlation function between the relaxation rates of a solution and the concentration of the paramagnetic substance [51]:

(1/*T*_1_ − 1/*T*_1*w*_) = *r*_1_ • [Gd] (1)

(1/*T*_2_ − 1/*T*_2*w*_) = *r*_2_ • [Gd] (2)

where *T*_1_ and *T*_2_ are the longitudinal and transverse relaxation times (in seconds), respectively, of the protons in the phantoms; *T*_1*w*_ and *T*_2*w*_ are the relaxation times (in seconds) of the protons in distilled water; and [Gd] is the concentration of the Gadolinium in mM.

First, we measured the *T*_1_ and *T*_2_ relaxation times of the protons in each of the phantoms. Then, we calculated the relaxation rate constants *R_i_* = 1/*T_i_*, where *T_i_*—is the longitudinal or transverse relaxation time. The obtained data was used to plot the dependence graphs of the values of the (1/*T_i_* − 1/*T_iw_*) on the Gd concentration in the samples.

The *T*_1_ measurements were carried out using a saturation recovery pulse sequence RAREVTR (Rapid Acquisition with Relaxation Enhancement with variable repetition time TR) in coronal projection with the following scan parameters: FOV (field of view): 8 cm × 8 cm, matrix: 32 × 32, in-plane resolution: 2.5 mm × 2.5 mm, number of slices: 1, slice thickness: 10 mm, number of averages: 2. For the phantoms with a Gd concentration of 25 mM, 12.5 mM and 6.25 mM, the TR values were set from 10 ms to 1000 ms and the echo time (TE) was 5 ms with a Rare-factor of 1—total scan time was 3 min and 54 s. For the other phantoms, the TR values were set from 125 ms to 15,000 ms and the effective echo time (TE_eff_) was 15 ms with a Rare-factor of 8—total scan time was 5 min and 55 s.

The *T*_2_ measurements were conducted using a spin echo pulse sequence MSME (multi-spin multi-echo) with the same geometric parameters that were applied for the *T*_1_ measurements. The difference was in the number of echoes, 64, and the number of averages, 1. For the phantoms with a Gd concentration of 25 mM, 12.5 mM and 6.25 mM, the TE values were set from 7.8 ms to 500 ms and the TR was 5000 ms—total scan time was 2 min. For the other phantoms, the TE values were set from 150 ms to 9600 ms and the TR was 12,000 ms—the total scan time was 2 min.

Twelve-week-old female *BALB/c Nude* mice were purchased from Pushino Animal Facility (Russia). The experimental procedures with mice were approved by the N.M. Emanuel Institute of Biochemical Physics Ethics Committee for the use of experimental animals and performed according to the guidelines of European Medicines Agency, Amsterdam, Netherlands. The mice (18–20 g) were inoculated subcutaneously with 10^6^ SKOV-3 cells in 100 µL DMEM. After subcutaneous tumor inoculation (day 0) at day 45 tumors reached sizes of ~800–1100 mm^3^. Two mice groups (*n* = 3) were used for the assessment of the MRI contrasting activity of double-targeted G4-DTPA-PEG-Pep immediately after injection; the control group was injected with Magnevist. The amount of drug injected was ~0.1 mL at a concentration of 15 mM (according to Gd). A spin echo pulse sequence RARE (Rapid Acquisition with Relaxation Enhancement) was used, and the scan parameters were as follows: FOV: 10 cm × 4 cm; matrix: 400 × 160; in-plane resolution: 0.25 mm × 0.25 mm; number of slices: 18; slice thickness: 1 mm; number of averages: 2; Rare-factor: 4; TR: 1300 ms; TE_eff_: 9.7 ms; scan time: 2 min and 36 s. To avoid the chest movement artifacts, the scan was produced using the respiratory gating monitoring system. Thus, the total scan time was twice as long and was about ~5 min.

The graphs were plotted using the Microsoft^®^ Excel^®^ 2013 program. The analysis of the in vivo MRI studies was carried out in the ImageJ v.1.51j software [52]. First, the MR images were normalized to the same noise, and then the signal intensity *I_s_* was calculated in the location of the tumor before *I_s_*_1_ and after the injection *I_s_*_2_ of the substance (Magnevist^®^ or G4-DTPA-PEG-Pep). The region of interest (ROI) was set as yellow circle, as shown in Figure 4b. The contrast assessment was performed by calculating the relative change in the signal intensity: *I_s_*_2_/*I_s_*_1_.

### 2.8. In Vivo and Ex Vivo Intratumoral Accumulation 

Three mice groups (*n* = 3) were used for the assessment of the intratumoral accumulation of Cy5.5-labeled G4-DTPA-PEG-Pep and two mono-targeted conjugates—G4-DTPA-PEG-KQ and G4-DTPA-PEG-IEL—4 h after injection of 0.2 mL of 100 nM solutions (according to Cy5.5 content); the control group was injected with non-targeted G4-DTPA-PEG. Tumors of the same groups were analyzed for the accumulation of conjugates 24 h after administration ex vivo. The visualization was performed using the IVIS Spectrum CT imaging system (Perkin Elmer, Hopkinton, Massachusetts USA), followed by Living Image software processing. During in vivo experiments, the mice were anesthetized with Isoflurane (Karizoo). 

### 2.9. Statistical Analysis

The data visualization and statistical analysis were performed in OriginPro (OriginLab Corporation, Northampton, MA, USA) and Excel (Microsoft Corporation, Redmond, WA, USA). The results are represented as mean ± standard deviation. One-way analysis of variance was applied to determine the significant difference. *p* < 0.05 was considered as significant.

## 3. Results and Discussion

### 3.1. Synthesis and Characterization of Peptides-Conjugated PAMAM-PEG

**Figure 1:**
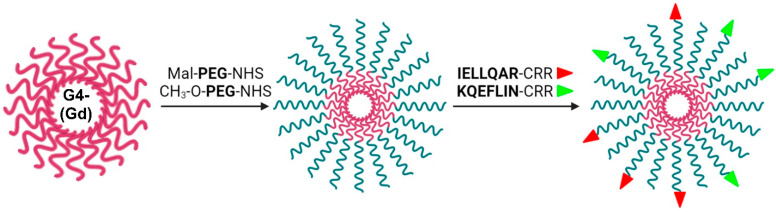
Schematic representation of the double-targeted G4-DTPA-PEG-Pep nanocarrier synthesis.

Figure 1 shows a schematic double-targeted G4-DTPA-PEG synthesis. The conjugate was synthesized by a covalent conjugation SCN-DTPA with dendrimer’s NH_2_-termini followed by a Gd chelation step. FITC or Cy-5.5 performed a label function for in vitro and vivo analysis. Further, we conjugated G4-DTPA with PEG arms containing methoxy moieties and maleimide followed by modification with cysteine-containing IELLQAR-CRR and KQEFLING-CRR peptides. CRR tags contained cysteine residue essential for maleimide conjugation and double arginine, which enhances peptides’ solubility. 

The final shell consisted of DTPA, peptides, and methoxy-PEG arms. These masked the positive surface charge, increased the hydrodynamic radius of PAMAM dendrimers, reduced the nonspecific cell membrane interaction and opsonization with plasma proteins, and enhanced the blood circulation time and in vivo tumor accumulation [53]. The tumor targeting ligands IELLQARCRR and KQEFLINGCRR were selectively conjugated with PEGs via maleimide residues and SH group of peptides, and provided an active targeting function to the final nanocarrier.

**Figure 2:**
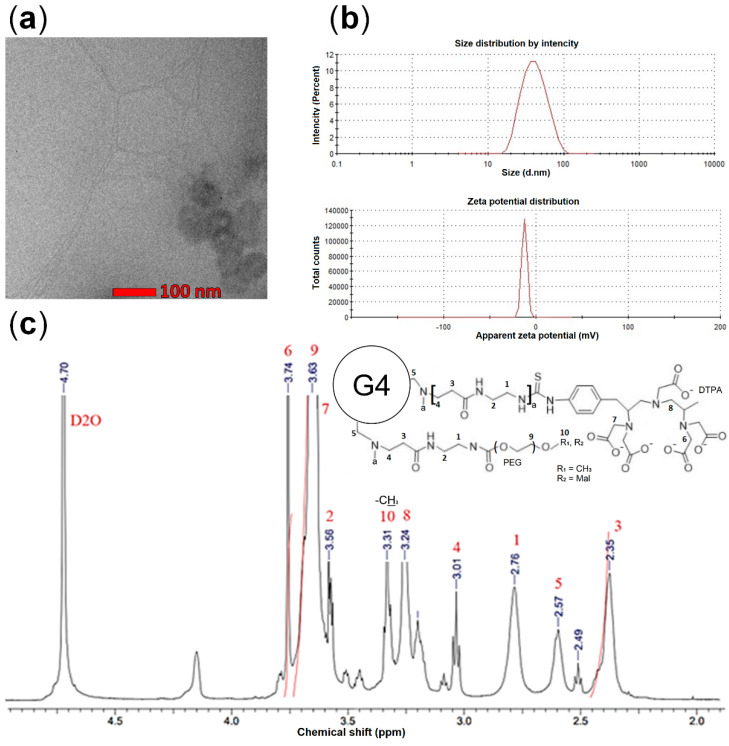
(**a**) TEM image of G4-DTPA-PEG-Pep (Bar 100nm). (**b**) G4-DTPA-PEG-Pep size and zeta potential. (**c**) G4-DTPA-PEG H^1^NMR spectra.

The G4-DTPA-PEG-Pep conjugate was characterized by a spherical shape (Figure 2a), a size of about 32.1 nm, and a negative zeta potential (−18.4 mV) (Figure 2b).

We applied ICP-MS analysis to evaluate the amount of Gd bound and H^1^NMR to evaluate the quantity of PEG conjugated to the dendrimer surface (Figure 2c). The major peak at 3.63 ppm corresponded to the methylene protons (CH_2_-O-CH_3_) of PEG; the peak at 6.75 ppm corresponded to maleimide protons (CH-CH) (R_2_); the peaks at 2.35, 2.57, 2.76, 3.01, and 3.56 ppm corresponded to the methylene protons of the dendrimer; the peaks at 3.24 and 3.74 ppm corresponded to the protons of DTPA (Figure 2c). We applied GHS/DTDP analysis to quantify the surface maleimide residues and analyze the amount of conjugated peptides.

According to the spectrophotometric and NMR analysis, the composition of conjugate consisted of 29 PEG (approximately 19 oxymethyl- and 10 maleimide-terminated PEG residues) and 18 DTPA residues per dendrimer. PEG quantity was evaluated based on the relative intensity ratio of the peaks at 3.63 and 2.35 ppm (Figure 2c), corresponding to the methylene protons (CH_2_CH_2_O) of PEG and the methylene protons (CH_2_CH_2_CONH) of dendrimers, respectively. The peptide conjugation demonstrated an approximately 60–80% efficiency according to the GHS/DTDP analysis: 1 µM of G4-DTPA-PEG bound approximately 4.1 µM of IELLQARCRR and 3.5 µM KQEFLINGCRR, regardless of the conjugation order.

### 3.2. Effects of Targeting Ligands on the Cellular Uptake

**Figure 3:**
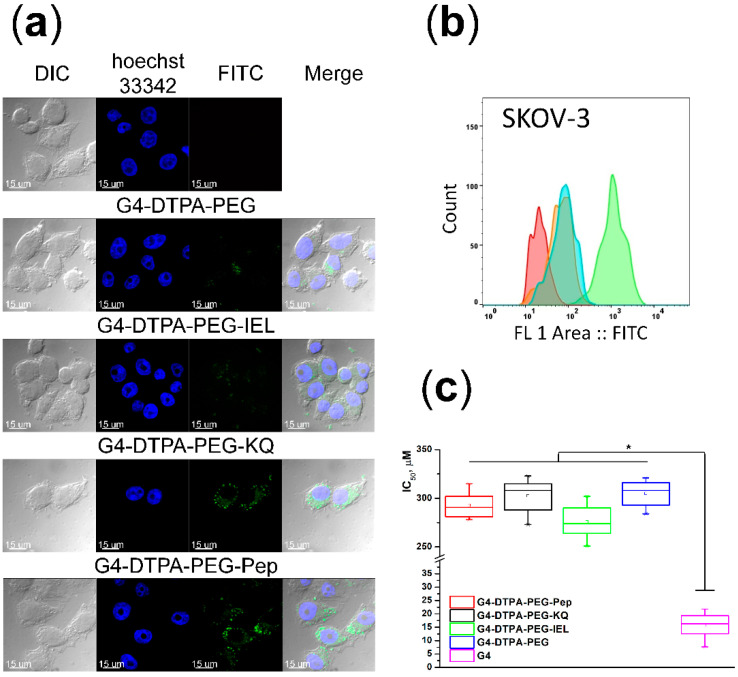
(**a**) CLSM images of SKOV-3 cells after incubation with FITC-labeled non-targeted G4-DTPA-PEG, mono-targeted G4-DTPA-PEG-IEL and G4-DTPA-PEG-KQ, and double-targeted G4-DTPA-PEG-Pep (bottom row) for 1 h; untreated control—upper row. Bar 15 µm. (**b**) Fluorescence intensity analysis of SKOV-3 cells after incubation with conjugates for 1 h: red—untreated control; green—double-targeted G4-DTPA-PEG-Pep; blue—non-targeted conjugate G4-DTPA-PEG; orange—mono-targeted conjugate G4-DTPA-PEG-IEL. (**c**) MTT assay results of the conjugates against SKOV-3 cells after incubation for 72 h. IC_50_ values represent the whisker plots (* *p* < 0.05).

We applied flow cytometry and CLSM to evaluate the cellular uptake of conjugates by AFP receptor-positive SKOV-3 cells. We used mono-peptide-conjugated G4-DTPA-PEG-KQ and G4-DTPA-PEG-IEL as the controls. Figure 3a displays the CLSM images of SKOV-3 cells after incubation with FITC-labeled G4-DTPA-PEG-Pep for 1 h. FITC fluorescence intensity revealed the ability of the targeted conjugate to bind and internalize in tumor cells, while cells incubated with counter-targeted G4-DTPA-PEG-Pep displayed much lower fluorescence levels, which may be evidence of the contribution of receptor-mediated endocytosis.

Flow cytometry analysis revealed a high level of internalization of G4-DTPA-PEG-Pep after incubation, while counter-targeted G4-DTPA-PEG-IEL and non-targeted G4-DTPA-PEG conjugates demonstrated low internalization, which coincided with the CLSM results (Figure 3b). 

Cytotoxicity analysis displayed a low level of activity of G4-DTPA-PEG-Pep against SKOV-3 cells in contrast with non-PEGylated G4 (Figure 3c).

### 3.3. In Vivo Biodistribution and Intratumoral Accumulation

**Figure 4:**
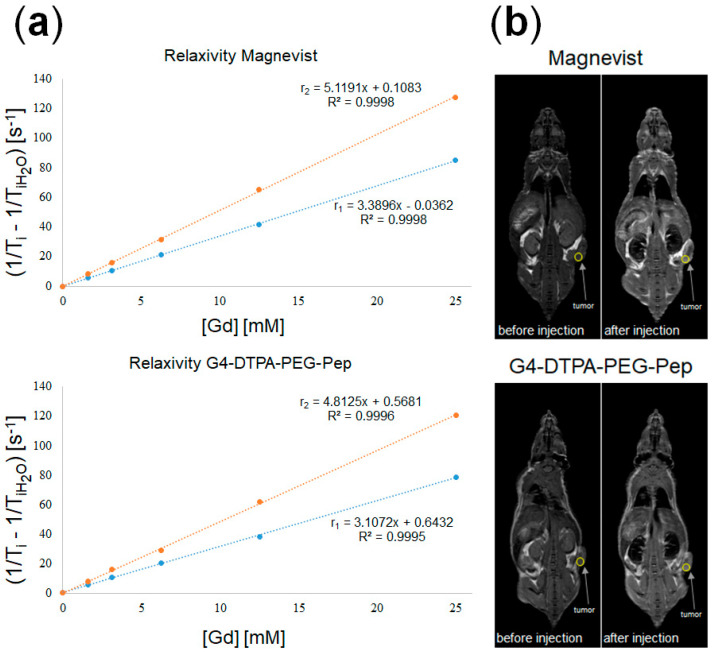
(**a**) In vitro r_1_ and r_2_ relaxivity of Magnevist (red line) and G4-DTPA-PEG-Pep (blue line). (**b**) In vivo mice MRI before and after Magnevist and G4-DTPA-PEG-Pep injection.

We evaluated the efficiency of G4-DTPA-PEG-Pep as a tumor-contrasting agent. The conjugate demonstrated a relaxivity comparable with that of Magnevist in vitro at the same Gd concentration: the r_1_ relaxivity was 3.39 for Magnevist and 3.11 for conjugate; the r_2_ relaxivity was 5.12 for Magnevist and 4.81 for conjugate (Figure 4a). Slightly lower values for conjugate could be explained by the hindered water accessibility to chelated Gd.

An in vivo contrasting evaluation, immediately after injection, also revealed properties of G4-DTPA-PEG-Pep comparable with those of Magnevist in SKOV-3 tumor-bearing *BALB/c Nude* mice (Figure 4b).

**Figure 5:**
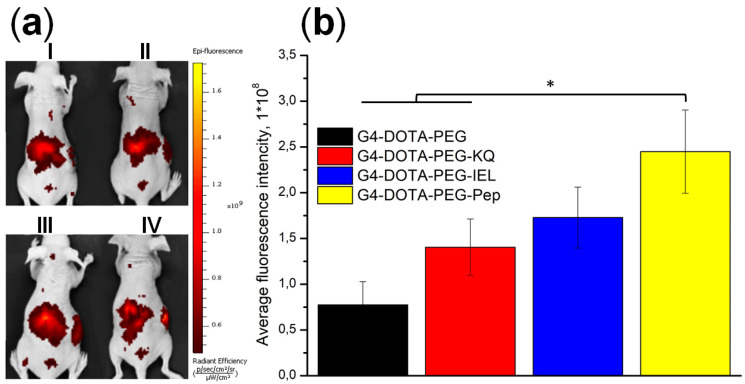
(**a**) In vivo images of SKOV-3 tumor-bearing *BALB/c Nude* 4 h after injection with Cy5.5-labeled G4-DTPA-PEG (I), G4-DTPA-PEG-KQ (II), G4-DTPA-PEG-IEL (III), or G4-DTPA-PEG-Pep (IV). (**b**) The conjugate accumulation in tumors of SKOV-3 tumor-bearing BALB/c Nude 24 h after administration (* *p* < 0.05).

Further, we assessed the biodistribution of G4-DTPA-PEG-Pep over a longer period. Briefly, we performed the visualization of SKOV-3 tumor-bearing *BALB/c Nude* mice 4 h after i.v. Cy5.5-labeled non-targeted G4-DTPA-PEG, mono-targeted G4-DTPA-PEG-KQ and G4-DTPA-PEG-IEL, and double-targeted G4-DTPA-PEG-Pep administration. All targeted conjugates demonstrated an effective intratumoral accumulation; the efficacy decreased in the following order: G4-DTPA-PEG-Pep > G4-DTPA-PEG-IEL > G4-DTPA-PEG-KQ > G4-DTPA-PEG (Figure 5). The lack of targeting moiety hindered the G4-DTPA-PEG-specific accumulation in tumors. The conjugates also revealed partial liver and bladder accumulation. The last observation coincided with MRI results that demonstrated an elevated kidney contrast immediately after administration, evidencing the presence and excretion of the low molecular weight fraction of G4-DTPA-PEG-Pep or non-conjugated DTPA-Gd.

Ex vivo fluorescence analysis 24 h after the conjugate administration confirmed the specific internalization of the conjugate in the tumor tissue (Figure 5b). Overall, despite some drawbacks, such as initial renal excretion and partial absorption by the liver, these results revealed a certain enhancement of the tumor-specific accumulation of the double-targeted conjugate over the mono-targeted one and proved the applicability of this strategy for tumor targeting.

## 4. Conclusions

Using a PAMAM dendrimer-based system decorated with two vectors for tumor targeting as a delivery system, we assessed whether double-targeting improves the accessibility of tumors for the transported drug compared to non-targeted or mono-targeted vehicles. We chose as the targets P-selectin, a marker of inflamed vasculature that is also characteristic of tumor tissue, and AFP receptor, which is a marker of a wide range of tumors, including SKOV-3 cells. Unfortunately, due to the distinct expression sites, it was impossible to evaluate the targeting properties of double-targeted conjugate in vitro in one experiment. However, double-targeted G4-DTPA-PEG-Pep demonstrated high internalization levels by SKOV-3 cells. In contrast, the same cells incubated with counter-targeted G4-DTPA-PEG-IEL revealed low internalization levels similar to those after incubation with non-targeted G4-DTPA-PEG conjugate. As expected, the PEGylated conjugates displayed low cytotoxicity. An in vitro r_1_ and r_2_ relaxivity assessment of G4-DTPA-PEG-Pep revealed similar values to those of commercially available Gd-chelate Magnevist. Immediately after injection, G4-DTPA-PEG-Pep demonstrated an insignificantly lower MRI contrasting ability in vivo compared to Magnevist; the conjugate also contrasted kidneys, which could be explained by a low molecular fraction or non-covalently bounded DTPA-Gd excretion. However, over longer periods, Cy5.5-labeled G4-DTPA-PEG-Pep displayed a clear tumor-associated localization. Thus, these results suggest that the double-targeted strategy, with the use of a proper nanocarrier, surface modification, and convenient vector selection, can be utilized to improve the specific anticancer drug delivery.

## Data Availability

Data are contained within the article.

## References

[1] Limeres M.J., Moretton M.A., Bernabeu E., Chiappetta D.A., Cuestas M.L. (2019). Thinking small, doing big: Current success and future trends in drug delivery systems for improving cancer therapy with special focus on liver cancer. Mater. Sci. Eng. C Mater. Biol. Appl..

[2] Pelaz B., Alexiou C., Alvarez-Puebla R.A., Alves F., Andrews A.M., Ashraf S., Balogh L.P., Ballerini L., Bestetti A., Brendel C. (2017). Diverse Applications of Nanomedicine. ACS Nano.

[3] Otto D.P., Otto A., de Villiers M.M. (2015). Differences in physicochemical properties to consider in the design, evaluation and choice between microparticles and nanoparticles for drug delivery. Expert Opin. Drug Deliv..

[4] Yang H., Zhao X., Zhang X., Ma L., Wang B., Wei H. (2018). Optimization of bioreducible micelles self-assembled from amphiphilic hyperbranched block copolymers for drug delivery. J. Polym. Sci. Part A Polym. Chem..

[5] Pooja D., Srinivasa R.T., Kulhari H., Kadari A., Adams D.J., Bansal V., Sistla R. (2020). N-acetyl-d-glucosamine-conjugated PAMAM dendrimers as dual receptor-targeting nanocarriers for anticancer drug delivery. Eur. J. Pharm. Biopharm..

[6] Tang H., Li C., Zhang Y., Zheng H., Cheng Y., Zhu J., Chen X., Zhu Z., Piao J.G., Li F. (2020). Targeted Manganese doped silica nano GSH-cleaner for treatment of Liver Cancer by destroying the intracellular redox homeostasis. Theranostics.

[7] Keshavarz M., Tan B., Venkatakrishnan K. (2017). Cell Selective Apoptosis Induced by Polymorphic Alteration of Self-Assembled Silica Nanowebs. ACS Appl. Mater. Interfaces.

[8] Wan X., Song L., Pan W., Zhong H., Li N., Tang B. (2020). Tumor-Targeted Cascade Nanoreactor Based on Metal-Organic Frameworks for Synergistic Ferroptosis-Starvation Anticancer Therapy. ACS Nano.

[9] Zhang F., Liu S., Zhang N., Kuang Y., Li W., Gai S., He F., Gulzar A., Yang P. (2020). X-ray-triggered NO-released Bi-SNO nanoparticles: All-in-one nano-radiosensitizer with photothermal/gas therapy for enhanced radiotherapy. Nanoscale.

[10] Faustova M., Nikolskaya E., Sokol M., Zabolotsky A., Mollaev M., Zhunina O., Fomicheva M., Lobanov A., Severin E., Yabbarov N. (2019). High-effective reactive oxygen species inducer based on Mn-tetraphenylporphyrin loaded PLGA nanoparticles in binary catalyst therapy. Free Radic. Biol. Med..

[11] Sokol M.B., Yabbarov N.G., Mollaeva M.R., Chirkina M.V., Balabanyan V.Y., Nikolskaya E.D. (2021). Development of the Composition and Technology for Obtaining Paclitaxel Nanoscale Formulation Consisting of a Conjugate of Polymer Particles with a Protein Vector Molecule. Drug Dev. Regist..

[12] Mollaeva M.R., Yabbarov N., Sokol M., Chirkina M., Mollaev M.D., Zabolotskii A., Seregina I., Bolshov M., Kaplun A., Nikolskaya E. (2021). Optimization, Characterization and Pharmacokinetic Study of Meso-Tetraphenylporphyrin Metal Complex-Loaded PLGA Nanoparticles. Int. J. Mol. Sci..

[13] Mollaeva M.R., Nikolskaya E., Beganovskaya V., Sokol M., Chirkina M., Obydennyi S., Belykh D., Startseva O., Mollaev M.D., Yabbarov N. (2021). Oxidative Damage Induced by Phototoxic Pheophorbide a 17-Diethylene Glycol Ester Encapsulated in PLGA Nanoparticles. Antioxidants.

[14] Sokol M.B., Nikolskaya E.D., Yabbarov N.G., Zenin V.A., Faustova M.R., Belov A.V., Zhunina O.A., Mollaev M.D., Zabolotsky A.I., Tereshchenko O.G. (2019). Development of novel PLGA nanoparticles with co-encapsulation of docetaxel and abiraterone acetate for a highly efficient delivery into tumor cells. J. Biomed. Mater. Res. B Appl. Biomater..

[15] Chen G., Roy I., Yang C., Prasad P.N. (2016). Nanochemistry and Nanomedicine for Nanoparticle-based Diagnostics and Therapy. Chem. Rev..

[16] Tietjen G.T., Bracaglia L.G., Saltzman W.M., Pober J.S. (2018). Focus on Fundamentals: Achieving Effective Nanoparticle Targeting. Trends Mol. Med..

[17] Mollaev M., Gorokhovets N., Nikolskaya E., Faustova M., Zabolotsky A., Zhunina O., Sokol M., Zamulaeva I., Severin E., Yabbarov N. (2019). Type of pH sensitive linker reveals different time-dependent intracellular localization, in vitro and in vivo efficiency in alpha-fetoprotein receptor targeted doxorubicin conjugate. Int. J. Pharm..

[18] Gonda A., Zhao N., Shah J.V., Calvelli H.R., Kantamneni H., Francis N.L., Ganapathy V. (2019). Engineering Tumor-Targeting Nanoparticles as Vehicles for Precision Nanomedicine. Med. One.

[19] Thomas O.S., Weber W. (2019). Overcoming Physiological Barriers to Nanoparticle Delivery-Are We There Yet?. Front. Bioeng. Biotechnol..

[20] Gao J.Q., Lv Q., Li L.M., Tang X.J., Li F.Z., Hu Y.L., Han M. (2013). Glioma targeting and blood-brain barrier penetration by dual-targeting doxorubincin liposomes. Biomaterials.

[21] Haense N., Atmaca A., Pauligk C., Steinmetz K., Marmé F., Haag G.M., Rieger M., Ottmann O.G., Ruf P., Lindhofer H. (2016). A phase I trial of the trifunctional anti Her2 × anti CD3 antibody ertumaxomab in patients with advanced solid tumors. BMC Cancer.

[22] Wang C.E., Yumul R.C., Lin J., Cheng Y., Lieber A., Pun S.H. (2018). Junction opener protein increases nanoparticle accumulation in solid tumors. J. Control. Release.

[23] Shu M., Gao F., Yu C., Zeng M., He G., Wu Y., Su Y., Hu N., Zhou Z., Yang Z. (2020). Dual-targeted therapy in HER2-positive breast cancer cells with the combination of carbon dots/HER3 siRNA and trastuzumab. Nanotechnology.

[24] Maeda H. (2010). Tumor-selective delivery of macromolecular drugs via the EPR effect: Background and future prospects. Bioconjug. Chem..

[25] Brighi C., Reid L., White A.L., Genovesi L.A., Kojic M., Millar A., Bruce Z., Day B.W., Rose S., Whittaker A.K. (2020). MR-guided focused ultrasound increases antibody delivery to nonenhancing high-grade glioma. Neurooncol. Adv..

[26] Kubo S., Takagi-Kimura M., Tagawa M., Kasahara N. (2019). Dual-vector prodrug activator gene therapy using retroviral replicating vectors. Cancer Gene. Ther..

[27] Mansoori B., Mohammadi A., Davudian S., Shirjang S., Baradaran B. (2017). The Different Mechanisms of Cancer Drug Resistance: A Brief Review. Adv. Pharm. Bull..

[28] Moro R., Tcherkassova J., Song E. (2005). A New Broad-Spectrum Cancer Marker. IVD Technology. http://citeseerx.ist.psu.edu/viewdoc/download?doi=10.1.1.605.495&rep=rep1&type=pdf.

[29] Moskaleva E.Y., Posypanova G.A., Koromyslova I.A., Shmyrev I.I., Krivonos A.V., Myagkikh I.V., Feldman N.B., Finakova G.V., Katukov V.Y., Luzhkov Y.M. (1996). In vivo antitumor activity of cytotoxic drugs conjugated with human alpha-fetoprotein. Tumor Target..

[30] Yabbarov N.G., Mollaev M.D., Zabolotskii A.I., Mazalev D.A., Gorokhovets N.V., Sokol M.B., Mollaeva M.R., Fomicheva M.V., Pshenichnikova A.B., Nikolskaya E.D. (2021). Obtaining and Purification of Recombinant Domain III of Human Alpha-Fetoprotein. Biotekhnologiya.

[31] Mollaev M., Zabolotskii A., Gorokhovets N., Nikolskaya E., Sokol M., Tsedilin A., Mollaeva M., Chirkina M., Kuvaev T., Pshenichnikova A. (2021). Expression of acid cleavable Asp-Pro linked multimeric AFP peptide in *E. coli*. J. Genet. Eng. Biotechnol..

[32] Severin S.E., Posypanova G.A., Katukov V.Y., Shmyrev I.I., Luzhkov Y.M., Gerasimova G.K., Zhukova O.S., Vorozhtsov G.N., Kaliya O.L., Lukyanets E.A. (1997). Antitumor activity of conjugates of the oncofetal protein alpha-fetoprotein and phthalocyanines in vitro. Biochem. Mol. Biol. Int..

[33] Posypanova G.A., Gorokhovets N.V., Makarov V.A., Savvateeva L.V., Kireeva N.N., Severin S.E., Severin E.S. (2008). Recombinant alpha-fetoprotein C-terminal fragment: The new recombinant vector for targeted delivery. J. Drug Target..

[34] Godovanny A.V., Savvateeva M.V., Sotnichenko A.I., Yabbarov N.G., Klimova O.V., Gnuchev N.V., Severin S.E. (2011). In vitro antitumor activity studying of AFP recombinat C-ending domain conjugate with cisplatine. Mol. Med..

[35] Zhang J., Alcaide P., Liu L., Sun J., He A., Luscinskas F.W., Shi G.P. (2011). Regulation of endothelial cell adhesion molecule expression by mast cells, macrophages, and neutrophils. PLoS ONE.

[36] Eichbaum C., Meyer A.S., Wang N., Bischofs E., Steinborn A., Bruckner T., Brodt P., Sohn C., Eichbaum M.H. (2011). Breast cancer cell-derived cytokines, macrophages and cell adhesion: Implications for metastasis. Anticancer Res..

[37] Blackwell J.E., Dagia N.M., Dickerson J.B., Berg E.L., Goetz D.J. (2001). Ligand coated nanosphere adhesion to E- and P-selectin under static and flow conditions. Ann. Biomed. Eng..

[38] Dickerson J.B., Blackwell J.E., Ou J.J., Shinde Patil V.R., Goetz D.J. (2001). Limited adhesion of biodegradable microspheres to E- and P-selectin under flow. Biotechnol. Bioeng..

[39] Kessner S., Krause A., Rothe U., Bendas G. (2001). Investigation of the cellular uptake of E-Selectin-targeted immunoliposomes by activated human endothelial cells. Biochim. Biophys. Acta.

[40] Natoni A., Macauley M.S., O’Dwyer M.E. (2016). Targeting Selectins and Their Ligands in Cancer. Front. Oncol..

[41] Chantarasrivong C., Ueki A., Ohyama R., Unga J., Nakamura S., Nakanishi I., Higuchi Y., Kawakami S., Ando H., Imamura A. (2017). Synthesis and Functional Characterization of Novel Sialyl LewisX Mimic-Decorated Liposomes for E-selectin-Mediated Targeting to Inflamed Endothelial Cells. Mol. Pharm..

[42] Ye Z., Zhang S., Liu Y., Wang S., Zhang J., Huang R. (2019). A Peptide Analogue of Selectin Ligands Attenuated Atherosclerosis by Inhibiting Monocyte Activation. Mediat. Inflamm..

[43] Hatakeyama S., Sugihara K., Shibata T.K., Nakayama J., Akama T.O., Tamura N., Wong S.M., Bobkov A.A., Takano Y., Ohyama C. (2011). Targeted drug delivery to tumor vasculature by a carbohydrate mimetic peptide. Proc. Natl. Acad. Sci. USA.

[44] Pippin C.G., Parker T.A., McMurry T.J., Brechbiel M.W. (1992). Spectrophotometric method for the determination of a bifunctional DTPA ligand in DTPA-monoclonal antibody conjugates. Bioconjug. Chem..

[45] Gouin S., Winnik F.M. (2001). Quantitative assays of the amount of diethylenetriaminepentaacetic acid conjugated to water-soluble polymers using isothermal titration calorimetry and colorimetry. Bioconjug. Chem..

[46] Diaz C., Guzmán J., Jiménez V.A., Alderete J.B. (2018). Partially PEGylated PAMAM dendrimers as solubility enhancers of Silybin. Pharm. Dev. Technol..

[47] Zhao D., Zhang Y., Wang Y., Xu C., Dong C., Li C., Ren S., Zhang W., Lu Y., Dai Y. (2014). Pharmacokinetics study of hemin in rats by applying ^58^Fe-extrinsically labeling techniques in combination with ICP-MS method. J. Pharm. Biomed. Anal..

[48] Pichler V., Mayr J., Heffeter P., Dömötör O., Enyedy É.A., Hermann G., Groza D., Köllenspeger G., Galanksi M., Berger W. (2013). Maleimide-functionalised platinum(IV) complexes as a synthetic platform for targeted drug de-livery. Chem. Commun..

[49] Dobrovolskaia M.A., Patri A.K., Simak J., Hall J.B., Semberova J., De Paoli Lacerda S.H., McNeil S.E. (2012). Nanoparticle size and surface charge determine effects of PAMAM dendrimers on human platelets in vitro. Mol. Pharm..

[50] Kumar P., Nagarajan A., Uchil P.D. (2018). Analysis of cell viability by the MTT assay. Cold Spring Harb. Protoc..

[51] Rohrer M., Bauer H., Mintorovitch J., Requardt M., Weinmann H.J. (2005). Comparison of magnetic properties of MRI contrast media solutions a different magnetic field strengths. Investig. Radiol..

[52] Schneider C.A., Rasband W.S., Eliceiri K.W. (2012). NIH image to Image J: 25 years of image analysis. Nat. Methods.

[53] Dąbkowska M., Łuczkowska K., Rogińska D., Sobuś A., Wasilewska M., Ulańczyk Z., Machaliński B. (2020). Novel design of (PEG-ylated)PAMAM-based nanoparticles for sustained delivery of BDNF to neurotoxin-injured differentiated neuroblastoma cells. J. Nanobiotechnol..

